# The exopolysaccharide–eDNA interaction modulates 3D architecture of *Bacillus subtilis* biofilm

**DOI:** 10.1186/s12866-020-01789-5

**Published:** 2020-05-14

**Authors:** Na Peng, Peng Cai, Monika Mortimer, Yichao Wu, Chunhui Gao, Qiaoyun Huang

**Affiliations:** 1 State Key Laboratory of Agricultural Microbiology, College of Resources of Environment, Huazhong Agricultural University, Wuhan, 430070 China; 2 Bren School of Environmental Science and Management and Earth Research Institute, University of California, Santa Barbara, California, 93106 USA

**Keywords:** Extracellular DNA (eDNA), Exopolysaccharide (EPS), *Bacillus subtilis*, Biofilm formation

## Abstract

**Background:**

Bacterial biofilms are surface-adherent microbial communities in which individual cells are surrounded by a self-produced extracellular matrix of polysaccharides, extracellular DNA (eDNA) and proteins. Interactions among matrix components within biofilms are responsible for creating an adaptable structure during biofilm development. However, it is unclear how the interactions among matrix components contribute to the construction of the three-dimensional (3D) biofilm architecture.

**Results:**

DNase I treatment significantly inhibited *Bacillus subtilis* biofilm formation in the early phases of biofilm development. Confocal laser scanning microscopy (CLSM) and image analysis revealed that eDNA was cooperative with exopolysaccharide (EPS) in the early stages of *B. subtilis* biofilm development, while EPS played a major structural role in the later stages. In addition, deletion of the EPS production gene *epsG* in *B. subtilis* SBE1 resulted in loss of the interaction between EPS and eDNA and reduced the biofilm biomass in pellicles at the air-liquid interface. The physical interaction between these two essential biofilm matrix components was confirmed by isothermal titration calorimetry (ITC).

**Conclusions:**

Biofilm 3D structures become interconnected through surrounding eDNA and EPS. eDNA interacts with EPS in the early phases of biofilm development, while EPS mainly participates in the maturation of biofilms. The findings of this study provide a better understanding of the role of the interaction between eDNA and EPS in shaping the biofilm 3D matrix structure and biofilm formation.

## Background

Bacterial biofilms are heterogeneous communities that exhibit a remarkable degree of spatiotemporal organization [1–3]. The spatial architecture of multicellular communities depends on the production of extracellular matrix, which is mainly composed of polysaccharides, proteins, and extracellular DNA (eDNA) [4, 5]. Extracellular DNA, as an important matrix component in biofilms [5, 6], can be used by bacteria for several vital functions; for example, as structural components of biofilms [7], nutrient sources [8], and a gene pool for horizontal gene transfer (HGT) [9]. The significance of eDNA in biofilm formation has been studied in *Pseudomonas aeruginosa* [6], *Staphylococcus epidermidis* [10], *Streptococcus pneumoniae* [11] and *Vibrio cholerae* [12]. In these studies, young biofilms were easily disturbed by DNase I treatment, but this treatment was not effective against aged biofilms. This loss of sensitivity to DNase I treatment suggests that in mature biofilms, either other extracellular matrix components complement or replace eDNA functions or that eDNA is shielded from enzymatic degradation when bound to other biofilm components.

Exopolysaccharide (EPS) is one of the major extracellular biofilm matrixes [13–15]. Interactions between EPS and eDNA have been investigated in some bacterial biofilms. In *Streptococcus mutans* biofilms, the interaction between eDNA and glucan results in the formation of filamentous structures that play an important role in connecting bacterial cells [16]. In the case of *P. aeruginosa*, eDNA and the exopolysaccharide Psl physically interact in biofilms to form the web of Psl–eDNA fibres, which function as a skeleton facilitating bacterial adhesion and growth [17]. Meanwhile, Psl can interact with genomic DNA from human neutrophils or strains of *S. aureus*, implying that *P. aeruginosa* can utilize genomic DNA from other organisms to form its own community [17]. Therefore, the eDNA–EPS interaction is important for the construction of biofilm architecture.

*Bacillus subtilis*, a gram-positive bacterium, produces a variety of biologically active compounds with a broad spectrum of activities against plant pathogens [18–23]. Due to the role of *B. subtilis* as a biocontrol agent in agricultural settings, a growing number of studies have focused on biofilm formation under natural and artificial conditions [18, 24–26]. Exopolysaccharide (EPS) is a key component in the *B. subtilis* matrix that promotes cell binding in structural biofilms [27]. It has been proposed that eDNA released by dead cells during the process of cannibalism in *B. subtilis* 168 could be related to matrix development [28–31]. On the other hand, eDNA released from *B. subtilis* 3610 during the stationary phase is not involved in biofilm establishment [32]. However, how interactions between eDNA and EPS modulate *B. subtilis* biofilm formation processes and architecture construction is less known.

This study focused on elucidating (1) the role of eDNA in the construction of the *B. subtilis* biofilm three-dimensional (3D) architecture and (2) the interaction between eDNA and EPS during biofilm formation. Here, we used confocal laser scanning microscopy (CLSM) and image analysis to investigate the role of eDNA during *B. subtilis* SBE1 biofilm structure formation. The *∆epsG* strain (deletion of one EPS production gene) was used to examine the role of EPS and its interaction with eDNA during biofilm development. To better understand the interactions of EPS and eDNA, isothermal titration calorimetry (ITC) was used to study the thermodynamics of the interactions between these two molecules.

## Results

### The role of extracellular DNA (eDNA) in the construction of the *B. subtilis* SBE1 biofilm three-dimensional (3D) architecture

**Fig. 1:**
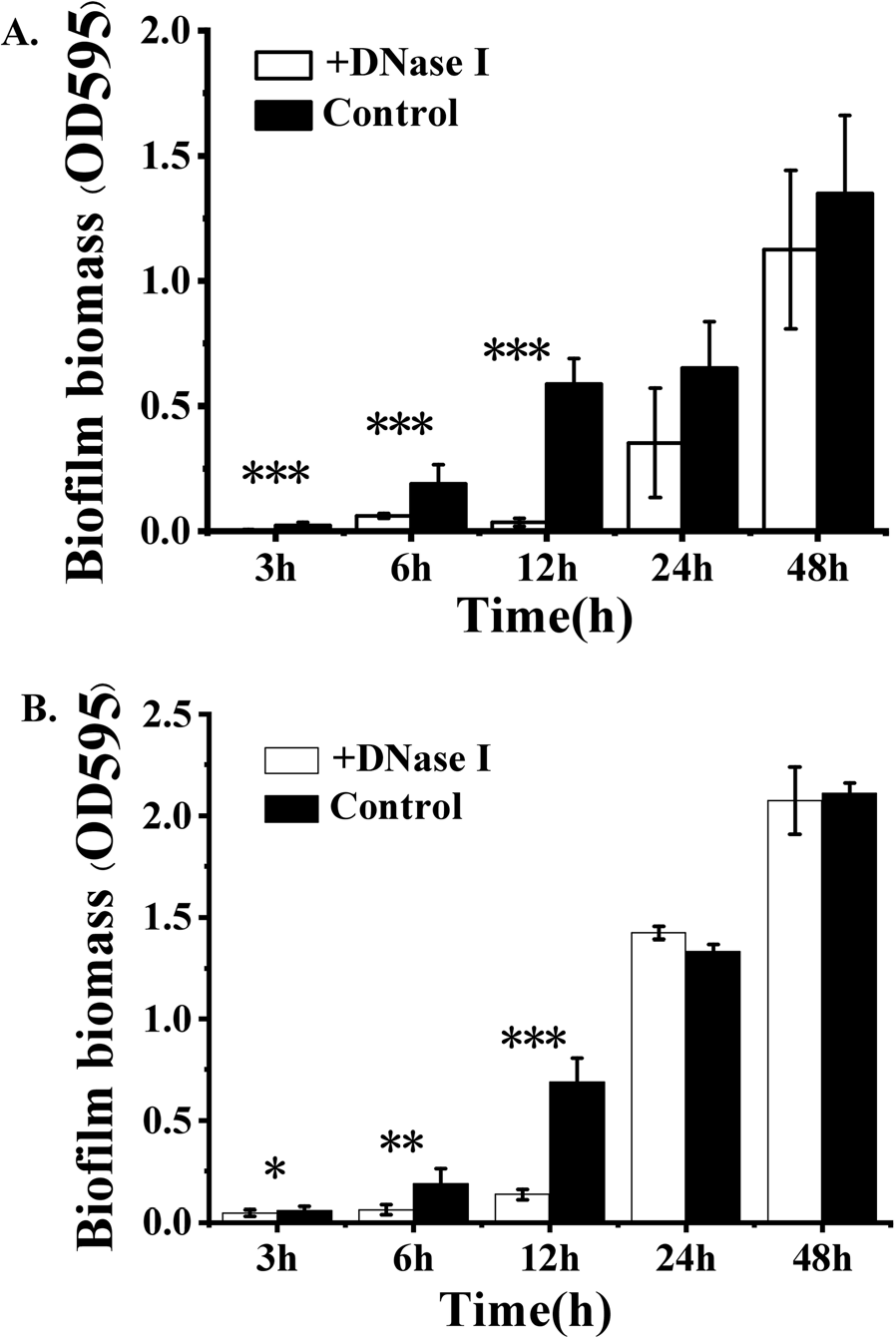
Effect of extracellular DNA (eDNA) removal on biofilm formation in microtiter plates. DNase I was either added at the beginning of the experiment (**a**) or after the biofilm had established. **b**. Biomass was quantified by using a crystal violet assay. White bars = DNase I treated, black bars = untreated control. The bars are means of five replicates, and the error bars represent standard deviations. ^*^P < 0.05, ^**^P < 0.01, ^***^P < 0.001 for comparisons of data obtained in the absence of DNase I and in the presence of DNase I

**Fig. 2:**
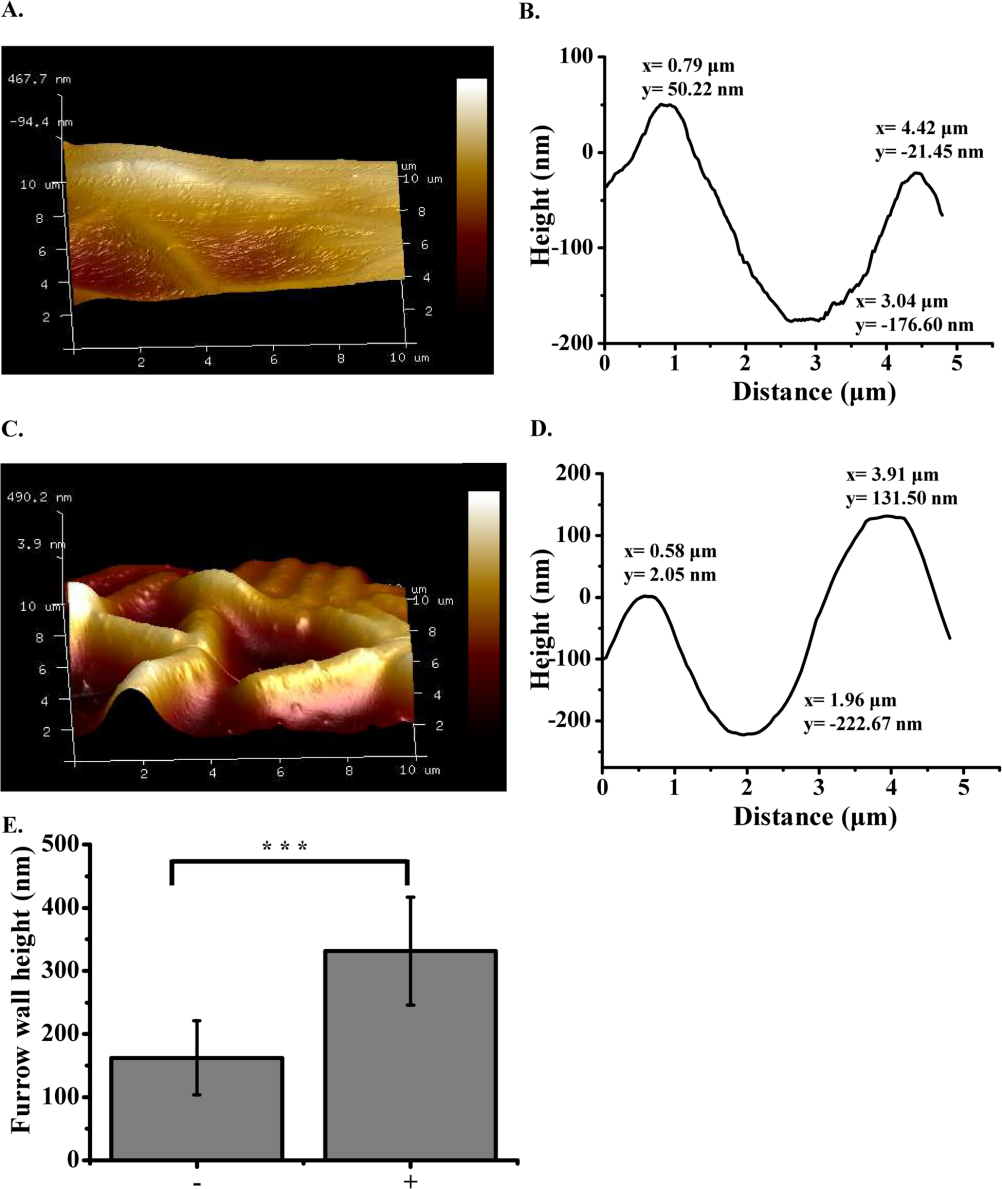
Atomic force microscopy (AFM) surface profiles of biofilms (12 h). 3D AFM images of biofilms cultured in the absence (**a**) and presence of DNase I (**c**). Scale is a relative color scale. A is scaled to 467.7 nm, and C is scaled to 490.2 nm. Measurements were taken between cells to generate a depth profile as shown in **b** and **d**. The y-axis scale is different for **b** and **d**. **e** Depths of furrows between cells from biofilms cultured in the absence (−, *n* = 10 from three AFM images) and presence (+, n = 10 from three AFM images) of DNase I. Error bars represent standard deviations. ^***^P < 0.001

In order to understand the contribution of eDNA in the biofilm formation of *B. subtilis* SBE1, the impact of DNase I on biofilm formation was tested using a static biofilm assay. The formation of biofilms grown for 3, 6, and 12 h with DNase I was clearly suppressed compared with the untreated control, based on crystal violet assay (3 h, *P* = 0.0020; 6 h, *P* = 0.0003; 12 h, *P* = 0.0000) (Fig. 1a). In contrast, biofilms grown with DNase I for 24 and 48 h were not significantly different from biofilms grown without DNase I (Fig. 1a). Furthermore, as shown in Fig. 1b, the biofilms that were 3, 6, and 12 h old when the DNase I treatments were initiated were dissolved (3 h, *P* = 0.420; 6 h, *P* = 0.0392; 12 h, *P* = 0.0005), whereas the biofilms that were 24 and 48 h old at the time of DNase I exposure were only affected to a minor degree. The biofilm treated with DNase I after 12 h was further characterized using atomic force microscopy (AFM) to measure the depth of the furrows generated (Fig. 2). In the absence of DNase I, furrows between cells were ~ 200 nm in depth (Fig. 2a, b and e). In the presence of DNase I, the furrows of the expanding biofilm were significantly deeper (up to 400 nm) than those formed in the absence of DNase I (Fig. 2c, d and e) (*P* < 0.001), suggesting that the gaps between cells in biofilms may be filled with eDNA. Therefore, eDNA may be an adhesion compound enabling cell-to-cell attachment, which initiates biofilm formation.

**Fig. 3:**
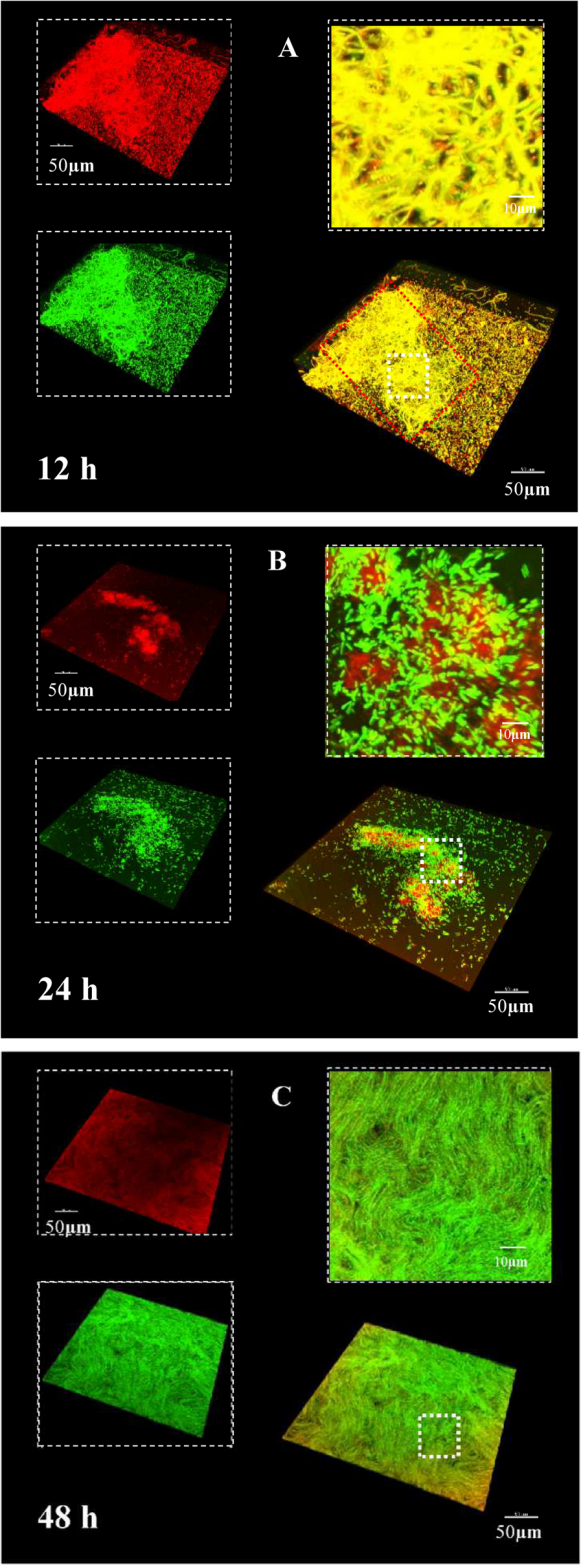
Dynamics of morphogenesis, three- dimensional (3D) architecture development and microbial population shifts of *B. subtilis* SBE1 biofilms. The biofilms stained with TOTO-1 for eDNA (green) and SYTO 60 for bacteria (red). Representative 3D rendering images of *B. subtilis* SBE1 biofilms at 12 h (**a**), 24 h (**b**) and 48 h (**c**). At the upper left of each panel, the two channels are displayed separately, while the merged image is displayed at the bottom right. A magnified (close-up) view of each small box depicted in the merged image is positioned in the upper right corner of each panel

**Fig. 4:**
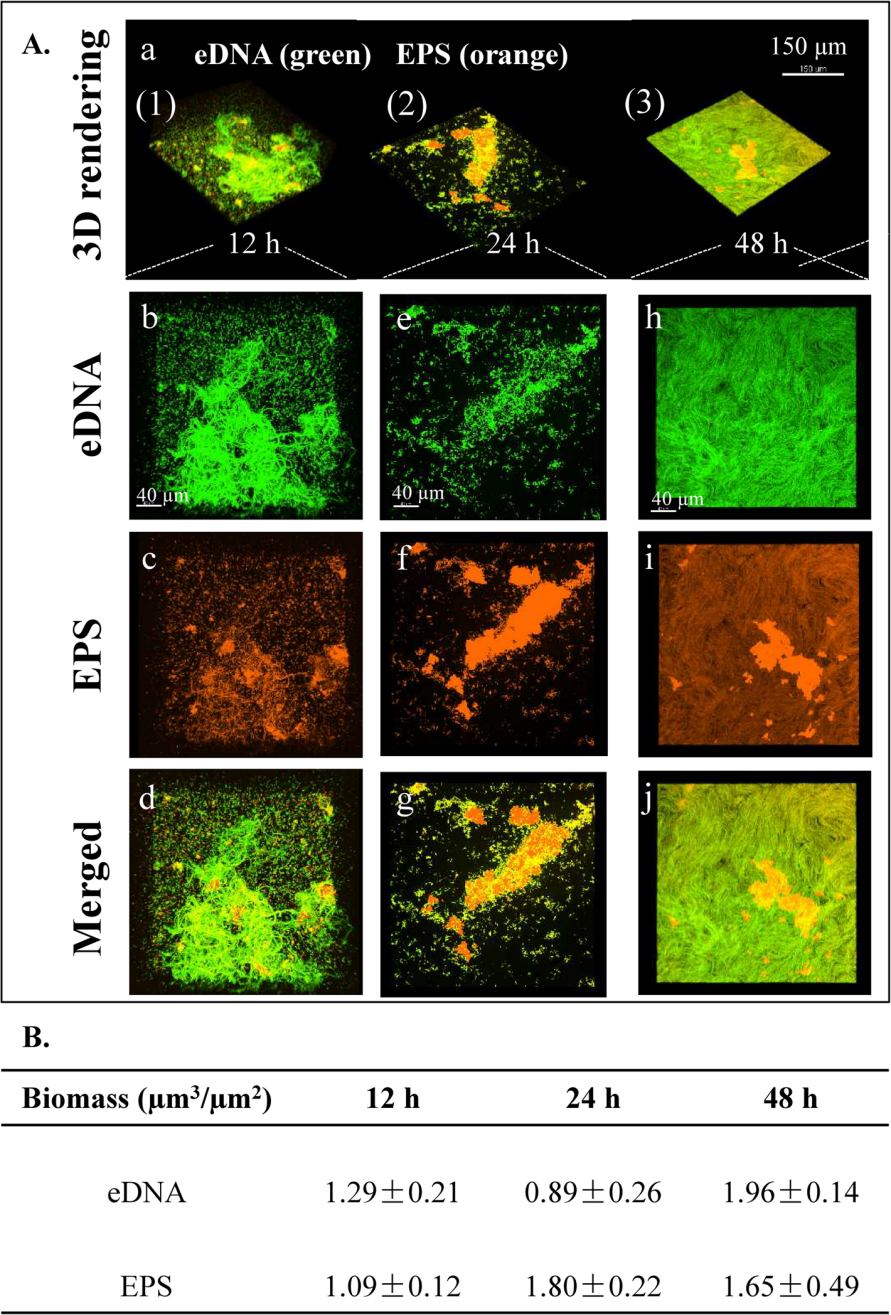
Structural arrangement between exopolysaccharide (EPS) and extracellular DNA (eDNA) during biofilm development of *B. subtilis* SBE1 and its EPS mutant (*∆epsG*). **A** Representative 3D renderings of *B. subtilis* SBE1 biofilms at 12, 24 and 48 h: (a) shows the dynamic evolution of biofilms over time. Panel (b-J) show cross sectional images of selected area for close-up views of structural organization of EPS (orange) and eDNA (green) during the development of biofilm matrix complex. **B** The biomass values of EPS and eDNA in the biofilms were calculated using Imaris. The data shown are mean values ± SD (*n* = 3)

To further understand how eDNA functions as a cell-cell adhesin during biofilm development, the construction of an eDNA matrix in a biofilm was examined over time. The formation and sequence of assembly of eDNA changed over time. After 12 h of biofilm development (Fig. 3a), the cells were densely packed in the eDNA matrix, forming a 3D biofilm structure (termed the eDNA-microcolony complex, highlighted with red box). After 24 h, the biofilm structures expanded in several dimensions with less eDNA-matrix enmeshed in and around the bacteria (Fig. 3b and c). Imaris analysis also showed that the content of eDNA in the biofilms was substantially reduced after 24 h (Fig. 4b). These results suggested that other matrix components (i.e., exopolysaccharides (EPS)) may complement eDNA as cell-cell adhesins when the biofilm becomes mature or perhaps that eDNA in mature biofilms interacts with other biomolecules that can protect the eDNA from DNase degradation. As shown in Fig. S1, in the following 24 h, more bacteria gathered in the biofilms. Meanwhile, the amount of eDNA increased again in the 48-h biofilm (Fig. 4), suggesting that eDNA may assist other matrix components during biofilm maturation.

### Exopolysaccharide (EPS) and eDNA colocalization in *B. subtilis* SBE1 3D biofilms

Exopolysaccharide (EPS) is an important extracellular biofilm matrix in *B. subtilis*. The spatial assembly of EPS and eDNA at each developmental stage of wild-type biofilms was examined at 12, 24 and 48 h by confocal laser scanning microscopy (CLSM) (Fig. 4). Figure 4Aa shows the 3D evolution of the EPS-eDNA interaction over time. The cross-sectional images at each time point are shown in Fig. 4Ab-j. At the 12-h time point, the biofilms contained considerable eDNA, which connected bacteria (Fig. 4A a (1)); at the 24-h time point, most eDNA was peripherally localized, and EPS was found to concentrate inside the biofilms (Fig. 4Aa (2)); at the 48-h time point, eDNA and EPS covered the entire structure (Fig. 4Aa (3)).

**Fig. 5:**
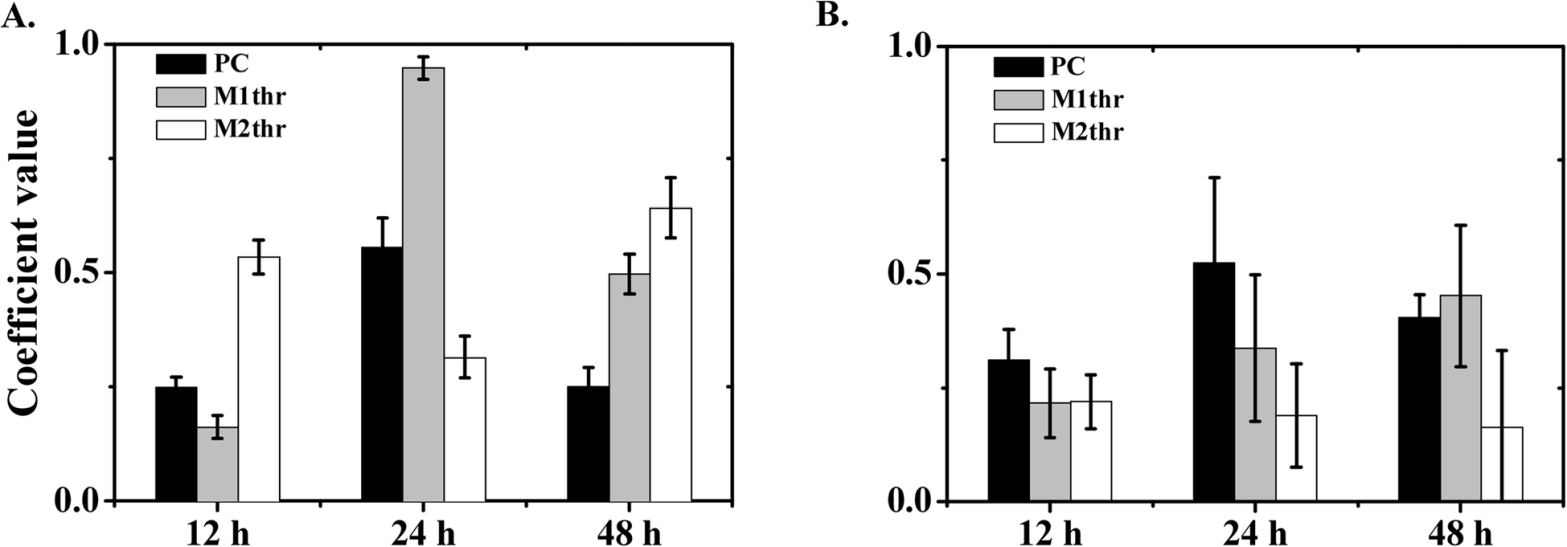
The analysis of extracellular DNA (eDNA)-exopolysaccharide (EPS) colocalization coefficients in *B. subtilis* SBE1 (**a**) and its EPS mutant (*∆epsG*) (**b**) biofilms. Columns showed the eDNA-EPS colocalization coefficients analyzed from seven images by three methods: Pearson’s correlation coefficient (PC), the thresholded Mander’s tM_1_ (M1thr) representing fraction of eDNA overlapping EPS, and tM_2_ (M2thr) representing fraction of EPS overlapping eDNA

This observation was supported by the quantitative colocalization analysis. As shown in Fig. 5, Pearson’s coefficient (PC) defines the quality of a linear relationship between two signals. Mander’s coefficients are based on Pearson’s correlation coefficient, and the average intensity values are obtained from the mathematical expression. The thresholded Mander’s coefficients were calculated by setting the threshold to the estimated value of background instead of zero. The analysis of seven image stacks by these methods showed the complete colocalization of eDNA-EPS in wild-type biofilms (Fig. 5a). The thresholded Mander’s tM_1_ (M1thr) indicated the fraction of eDNA (TOTO-1 signal, green) overlapping with EPS (ConA signal, orange), and tM_2_ (M2thr) indicated the fraction of EPS overlapping eDNA. The PC (black bar) will tend to 0 when random noise is added to complete colocalizing structures. At 12 h, a wider distribution of eDNA was observed in the intercellular space compared to EPS. Most of the EPS overlapped with the eDNA (M1thr < 0.5, M2thr > 0.5). At 24 h, most of the eDNA overlapped with the EPS (M1thr > 0.5, M2thr < 0.5). At 48 h, the eDNA and EPS were completely colocalized (M1thr > 0.5, M2thr > 0.5). This quantitative analysis was consistent with the CLSM observations, suggesting that eDNA was cooperative with EPS in early stages, while EPS might play a larger role in the later stages of *B. subtilis* SBE1 biofilm development.

### Spatial assembly of EPS and eDNA in biofilms of the *B. subtilis* SBE1 *eps*G mutant

**Fig. 6:**
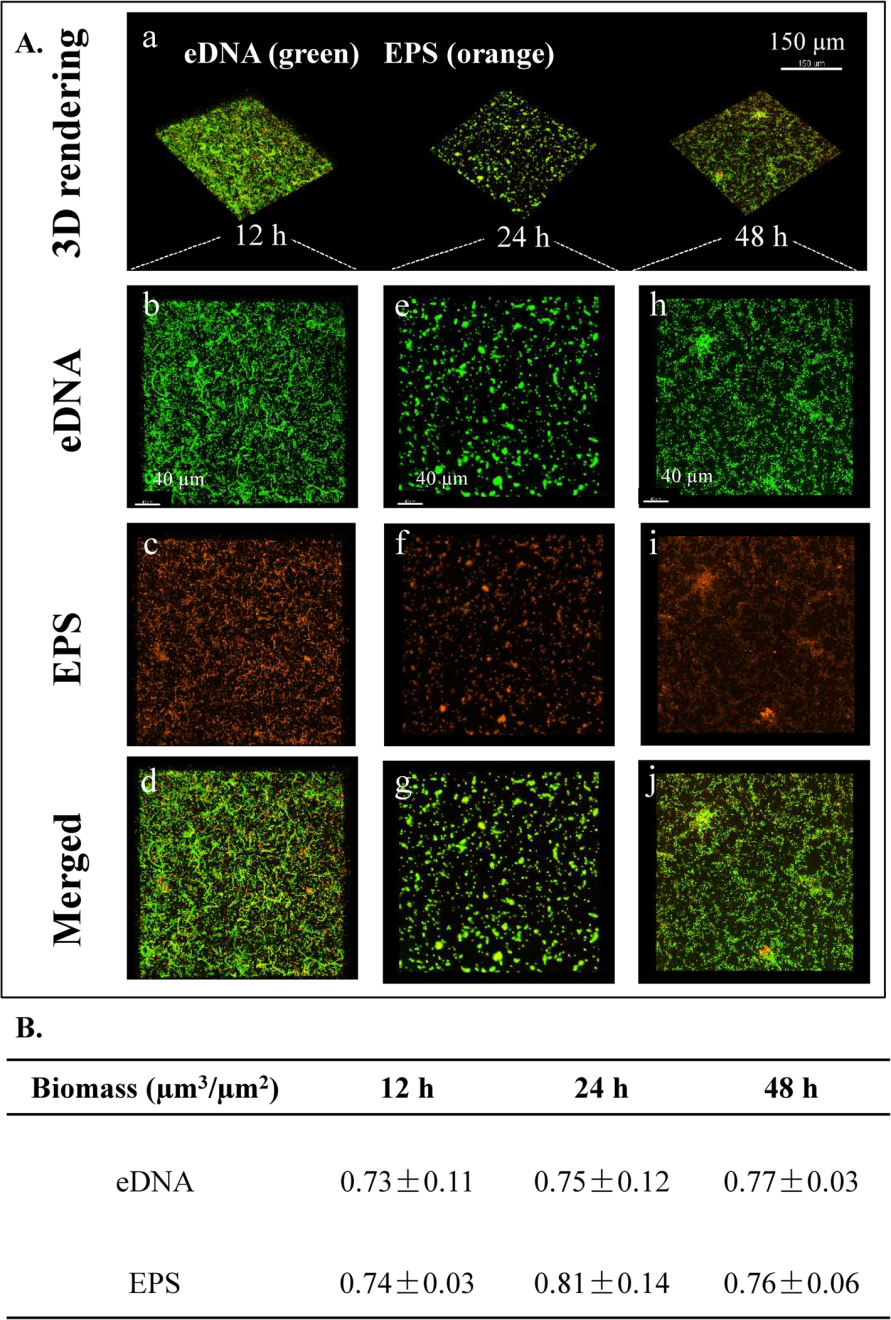
Structural arrangement between exopolysaccharide (EPS) and extracellular DNA (eDNA) during biofilm development of EPS mutant (*∆epsG*). **A** Representative 3D renderings of *B. subtilis* SBE1 biofilms at 12, 24 and 48 h: (a) shows the dynamic evolution of biofilms over time. Panel (b-J) show cross sectional images of selected area for close-up views of structural organization of EPS (orange) and eDNA (green) during the development of biofilm matrix complex. **B** The biomass values of EPS and eDNA in the biofilms were calculated using Imaris. The data shown are mean values ± SD (*n* = 3)

To further confirm the spatial assembly of EPS and eDNA during the development of *B. subtilis* SBE1 biofilms, an EPS defective mutant was constructed. The genome of *B. subtilis* SBE1 contains homologous *epsA-O* genes (the genetic locus for the *epsG* gene is as follows: open reading frame Query_1049240 [*epsG*]). As shown in Fig. S2, the *∆epsG* mutant produced significantly less cell-associated EPS than wild-type cells. The deletion of the EPS production gene *eps*G in *B. subtilis* SBE1 also resulted in a reduction in biofilm biomass in pellicles at the air-liquid interface (Fig. S1). Then, the spatial assembly of EPS and eDNA at each developmental stage of *∆epsG* mutant biofilms was also examined at 12, 24 and 48 h by CLSM (Fig. 6). Quantitative image analysis showed that there was a substantial reduction in the contents of both eDNA and EPS in the biofilms of the *∆epsG* mutant compared to wild-type *B. subtilis* SBE1 (Figs. 4 and 6). And quantitative colocalization analysis showed that eDNA colocalized with EPS in *B. subtilis* SBE1 pellicles but not in *∆epsG* strain pellicles. In addition, the eDNA and EPS in *∆epsG* mutant biofilms exhibited partial colocalization (12 h, 24 h) (M1thr < 0.5, M2thr < 0.5) (Fig. 5b). These results confirmed that eDNA interacts with EPS during biofilm development.

The colocalization of eDNA and EPS in *B. subtilis* SBE1 pellicles suggested the potential physical interaction between these two components. Isothermal titration calorimetry (ITC) is an important technique to study the thermodynamics of molecular interactions. The interaction between DNA and EPS could be determined by entropy changes (Δ*H*) because of the binding between them. Consistently, isothermal titration calorimetry results showed that the total heat produced from DNA from *B. subtilis* SBE1, *B. subtilis* 3610, *Shewanella oneidensis* MR1 and *P. putida* KT2440 binding to EPS was (1.5 ± 0.2) × 10^− 4^, (2.4 ± 0.3) × 10^− 4^, (9.5 ± 0.5) × 10^− 4^ and (8.6 ± 0.4) × 10^− 4^ KJ (*n* = 3), respectively (Fig. S3E). The calculated binding enthalpy (the binding heat per DNA molecule) of EPS was 222.72 ± 26.34, 162.77 ± 17.82, 338.46 ± 45.23 and 398.50 ± 38.67 kJ/mol (*n* = 3) for *B. subtilis* SBE1, *B. subtilis* 3610, *Shewanella oneidensis* MR1 and *P. putida* KT2440, respectively (Fig. S3F), indicating that the interaction between DNA from MR1 and KT2440 and EPS is more exothermic than DNA from SBE1 and 3610. However, EPS of *B. subtilis* SBE1 can interact with DNA from these bacteria, which might enable *B. subtilis* SBE1 cells to bind eDNA from these bacteria, leading to biofilm formation.

## Discussion

Interactions among matrix components within biofilms are responsible for creating an adaptable structure during biofilm development. However, how interactions between extracellular DNA (eDNA) and exopolysaccharide (EPS) modulate *B. subtilis* biofilm formation processes and architecture construction is less known. In this study, we focused on elucidating (1) the role of eDNA in the construction of the *B. subtilis* biofilm three-dimensional (3D) architecture and (2) the interaction between eDNA and EPS during biofilm formation.

We found that *B. subtilis* SBE1 biofilms were dissolved when the DNase I treatments were initiated, whereas the biofilms after 24 h at the time of DNase I exposure were only affected to a minor degree. Young biofilms are easily removed by DNase, but DNase treatment is not effective once the biofilm has aged past a certain point. Such a transition has been documented for, for example, *S. epidermidis* (at 12 h) [10], *P. aeruginosa* (at 80 h) [6], and *Vibrio cholera* (at 72 h) [12]. The use of DNase for biofilm removal is effective but dependent on the age of the biofilm. What causes the resistance of the biofilm to DNase remains to be explored, but this temporary sensitivity suggests either that other extracellular matrix components replace or complement eDNA within the mature biofilm or that eDNA is bound by another component that shields it from enzymatic degradation. Similar results have been observed in *Listeria monocytogenes* that peptidoglycan, as an additional essential component, is required for DNA-dependent biofilm development [33]. eDNA has been shown to play an important role in cell-to-cell interconnection during early *B. subtilis* SBE1 biofilm formation. It has been previously reported that cells can interact during the biofilm accumulation phase of *S. aureus* through recycled cytoplasmic proteins, which can be linked by eDNA [34]. Thus, another biofilm component may interact with eDNA to stabilize biofilm structure.

Exopolysaccharide is an important extracellular biofilm matrix in *B. subtilis*. The colocalization of eDNA and EPS observed in native extracellular matrix provided evidence for direct interactions between eDNA and EPS in *B. subtilis* SBE1 biofilms. Previous studies have been reported that the major EPS component of all *B. subtilis* biofilms is synthesized by the products of the 15-gene operon *eps A-O* (referred to as the *eps* operon) [27, 35–38]. The molecular structure of EPS has yet to be elucidated. To date, only a subset of EPS genes has been studied individually. EpsA and EpsB act as tyrosine kinase modulators and tyrosine kinases, respectively, and both are required for biofilm formation [37]. EpsE is a bifunctional protein that coordinates the production of EPS with the cessation of motility [38]. EpsG is a protein that is presumably involved in EPS polymerization [27]. Among these genes, the deletion of *eps*G could prevent surface-adhered biofilm formation even in the ∆*sip*W suppressor strain [39]. The above-described results indicate that eDNA may cooperate with EPS, which promotes cell-cell adhesion during early biofilm development. To confirm this, the *∆epsG* mutant was constructed to weaken the function of surface adhesion of EPS during biofilm formation. eDNA colocalized with EPS in *B. subtilis* SBE1 pellicles but not in *∆epsG* strain pellicles. There was also a substantial reduction in the contents of both eDNA and EPS in the biofilms of the ∆*eps*G mutant compared to wild-type *B. subtilis* SBE1. Biofilms formed by the ∆*eps*G mutant contained eDNA that did not colocalize with EPS in the biofilms. A similar pattern has been observed in *P. aeruginosa* PAO1. The Pel and Psl polysaccharides contribute to eDNA release and distribution during PAO1 biofilm development. Biofilms formed by the PAO1∆*pel*A mutant contained eDNA in the inner parts of microcolony structures. Biofilms formed by the PAO1∆*psl*BCD mutant contained a small amount of eDNA close to the substratum of biofilms [40]. It is possible that *eps*G may be involved in eDNA release and distribution during *B. subtilis* SBE1 biofilm formation.

Extracellular DNA interacts with EPS in the early phases of biofilm development, while EPS played a major structural role in the later stages. This transition of the role of eDNA from initial construction of the 3D extracellular matrix to matrix microaggregation is similar to the role of eDNA and lipoteichoic acid (LTA) in biofilms of *Streptococcus mutants* [41]. The colocalization of eDNA and EPS in *B. subtilis* SBE1 pellicles suggested the potential physical interaction between these two components. Previous work has used Isothermal titration calorimetry (ITC) to study the molecular interaction between protein and lipid polysaccharide (LPS) [42]. The interaction between DNA from *S. oneidensis* MR and *P. putida* KT2440 and EPS is more exothermic than DNA from *B. subtilis* SBE1 and *B. subtilis* 3610. This selectivity between DNA and EPS may be driven by a small energy difference of the interactions, such as electrostatic and van der Waals forces [43]. However, EPS of *B. subtilis* SBE1 can interact with DNA from *S. oneidensis and Pseudomonas putida* which also inhabit the soil. These species that commonly share soil ecosystems with *B. subtilis*, maybe associated in multispecies biofilms. In the bulk soil, bacteria are found in patches or microcolonies containing low cell numbers, often composed of different bacterial species [44, 45]. When exposed to nutrient sources, these microcommunities have the potential to develop into multispecies biofilms with high bacterial density [45]. The first step of the succession in an early multispecies biofilm based on the ability of surface/cell-cell attachment of soil bacteria [46]. Thus, EPS of *B. subtilis* can interact with DNA from these bacteria, which might enable *B. subtilis* cells to bind eDNA from these bacteria, initiating the biofilm formation.

## Conclusions

**Fig. 7:**
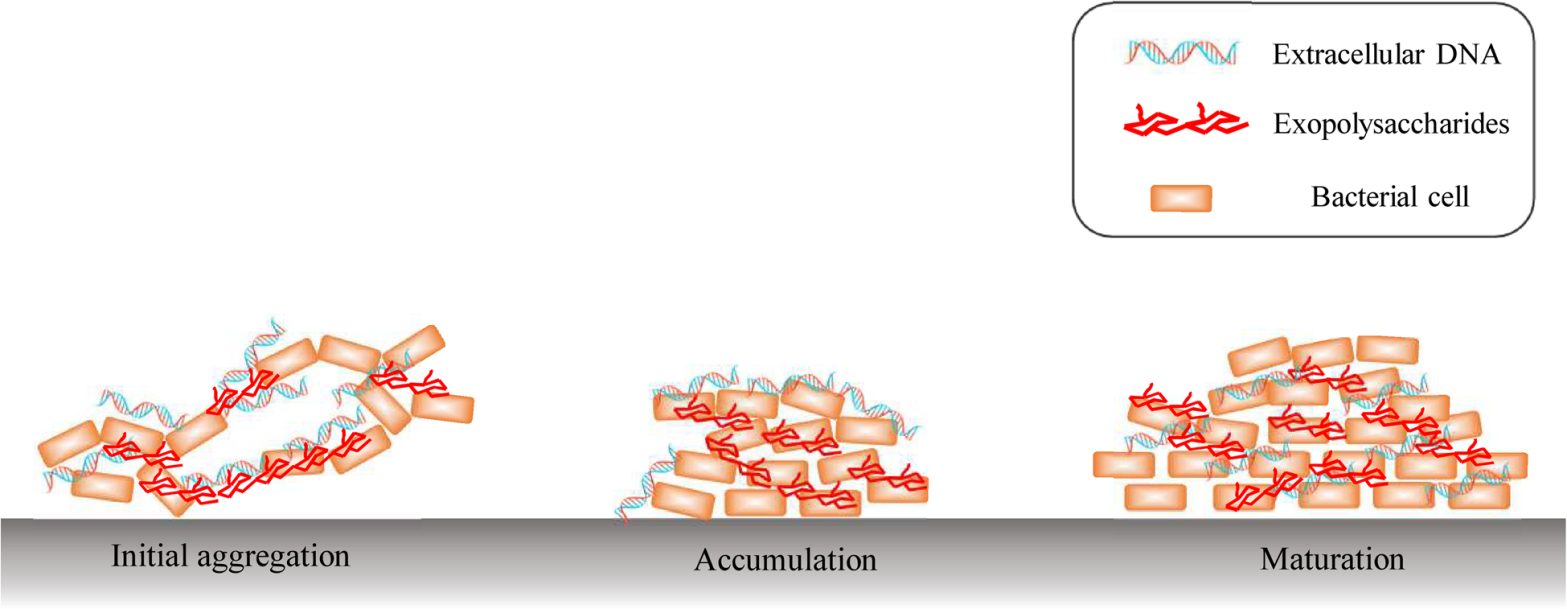
Proposed model for how extracellular DNA (eDNA)-exopolysaccharide (EPS) interaction modulates 3D architecture of *B. subtilis* SBE1 biofilm. Complex 3D biofilm forms in the following sequence: (1) initial aggregation, bacterial cells are connected and bridged by eDNA and EPS; (2) accumulation, a core of EPS-enmeshed bacterial cells is formed to provide a supporting framework; and (3) maturation, bacterial cells divide and accumulate, and EPS and DNA are evenly distributed in the biofilm

Extracellular DNA (eDNA) and exopolysaccharide (EPS), two essential matrix components of the *B. subtilis* SBE1 biofilm, cooperate by physically interacting in bacterial biofilms. Over time, the biofilm three-dimensional (3D) structures become interconnected through surrounding eDNA and EPS. eDNA interacts with EPS in the early phases of biofilm development, while EPS mainly participates in the maturation of biofilms. Based on our research, we proposed a model to describe how the eDNA-EPS interaction mediates the construction of the complex 3D biofilm architecture and establishes spatial heterogeneities in *B. subtilis* SBE1. Complex 3D biofilms form in the following sequence: (1) initial aggregation, bacterial cells are connected and bridged by eDNA and EPS; (2) accumulation, a core of EPS-enmeshed bacterial cells is formed to provide a supporting framework; and (3) maturation, bacterial cells divide and accumulate, and EPS and DNA are evenly distributed in the biofilm (Fig. 7). The interaction between eDNA and EPS plays a vital role in the construction of 3D biofilm architecture. In addition, the eDNA-EPS interaction might increase the survival of *B. subtilis* SBE1 in different environments by allowing eDNA from other microbial species to act as a scaffold on which a community can grow.

## Methods

### Bacteria strains and cultivation conditions

*Bacillus subtilis* SBE1, a wild-type soil isolate, was obtained from the State Key Laboratory of Agricultural Microbiology, Huazhong Agriculture University (Wuhan, China). *B. subtilis* SBE1 has been studied in our previous work where we showed that it can form biofilms in the presence of soil clay minerals and iron oxides [47]. This strain has been deposited in the China Centre for Type Culture Collection (CCTCC), and the accession number is CCTCC AB 2018210. The whole genome sequences have been deposited at DDBJ/ENA/GenBank under accession number QPGT01000000. The *∆epsG* mutant defective for exopolysaccharide (EPS) polymerization was constructed by using homologous recombination as previous described [48]. *Escherichia coli* strains DH5α obtained from the State Key Laboratory of Agricultural Microbiology were used for standard DNA manipulations [49]. The kanamycin resistance gene in pDG780 plasmid [49] was inserted into integration sites (*epsG*) in the genome of *B. subtilis* after inducing electroporation transformation [48]. The primers designed in this study are 1-F:CTAGTCTAGACGCCCCAAATGGGCAGGC, 1-R:CCGGAATTCC-GGTCATGGTCCTTTTCC; 3-F:CCGCTCGAGTTTATGCACGAGGAGCCG, 3-R:CGGGGTACCGAAGCTGAAAAACTGATC. The relative amounts of bacterial extracellular carbohydrates were estimated by the phenol-sulfuric acid method (see details in the supplementary material) [50]. Planktonic cultures were maintained on Luria–Bertani (LB) medium (10 g of tryptone, 5 g of yeast extract, and 10 g of NaCl per litre of broth) at 37 °C. *B. subtilis* SBE1 biofilms were cultivated at 37 °C in minimal salt glycerol glutamate (MSgg) medium [51].

### DNase I treatment of biofilm

The role of extracellular DNA (eDNA) in *B. subtilis* biofilm formation was investigated by adding DNase I (100 Kunitz units per mL) (Sigma-Aldrich, Steinheim, Germany) to an inoculum of *B. subtilis* to degrade the eDNA produced during growth. Briefly, after incubation (see Strains and cultivation conditions above), bacteria were resuspended in MSgg medium [51] and diluted to an OD_600_ of 0.05. Next, 200 μL of the bacterial suspension was added to each well of a 96-well microtiter plate (Costar, Corning Incorporated, Corning, NY) and incubated at 37 °C without shaking. After 3, 6, 12, 24, 48, and 72 h, the supernatant from each well was removed, and then the biofilms (pellicles) were washed twice with 1 mL of sterile 0.9% NaCl. In addition, 10 μL of DNase I (final concentration: 100 Kunitz units per mL) was added either at the beginning of the experiment or after the biofilm was established. The biofilm biomass was quantified using crystal violet assays [52]. For each analysis, 1% w/v crystal violet solution (200 μL) was added to eight replicate wells, incubated for 10 min, and rinsed twice with 200 μL of sterile distilled water. The crystal violet in the residual biofilm was dissolved in 200 μL of absolute ethanol. Then, the OD at 595 nm was measured using a plate reader (PerkinElmer, Waltham, USA).

### Atomic force microscopy (AFM)

The topography of the biofilms with and without DNase I treatment (12 h) was investigated using a MultiMode 8 AFM with a NanoScope V controller (Bruker). The scanning modes used were as follows: 1) ScanAsyst mode using ScanAsyst-Air cantilevers with 0.4 N m^− 1^ nominal spring constant (Bruker) and 2) tapping mode using RTESP cantilevers with 40 N m^− 1^ nominal spring constant (Bruker) [53]. A scan size of 10 × 10 μm was used. Images were processed and analysed using NanoScope Analysis (Bruker).

### Confocal image acquisition and analysis

The air-liquid interface biofilms were grown in 20 mm flat bottom cell culture dishes (Costar, Corning Incorporated, Corning, NY). DNase I (100 Kunitz units per mL) was added to the glass chamber during inoculation. For confocal laser scanning microscopy (CLSM) observation, buffer was gently removed from the glass chambers to allow the pellicles to drop onto the glass bottom [53]. The biofilms were stained with a Live/Dead™ Bacterial Viability Kit (Bac Light™, Molecular Probes, Invitrogen). The arrangement of the biofilm matrix was determined by direct incorporation of fluorescent labels during synthesis of the exopolysaccharide (EPS) and extracellular DNA (eDNA) matrix, which allowed the examination of the three-dimensional (3D) structure within intact biofilms [15, 54]. The labelling of EPS and eDNA matrix was performed after biofilms were developed. Extracellular DNA in biofilms was labelled by TOTO-1 nucleic acid stain (cell impermeable, 514/533 nm; Molecular Probes) and PI nucleic acid stain (cell impermeable, 535/615 nm; Molecular Probes). The exopolysaccharide matrix was labelled by ConA (α-polysaccharides) (590/617 nm; Molecular Probes) [55]. The bacteria in biofilms were labelled by SYTO 9 nucleic acid stain (cell permeable, 485/535 nm; Molecular Probes) and SYTO 60 nucleic acid stain (cell permeable, 652/678 nm; Molecular Probes). The imaging was performed using an Olympus FV 1000 monophoton laser scanning microscope (Olympus, Tokyo, Japan) equipped with a 40× (0.95 numerical aperture) objective lens.

The confocal images were analysed by using software to visualize and quantify the bacterial cells, EPS and eDNA within intact biofilms. Imaris 7.4.2 (Bitplane AG, Zurich, Switzerland) was used to rebuild each structural component (bacteria, EPS and eDNA) within the biofilms to visualize the 3D architecture and morphology. Quantitative characterization of each structural component within the 3D biofilm images was performed as reported previously [15, 56]. The images were imported to JACoP (Fabrice P. Cordelieres, Institut Curies, Orsay, France), a plugin for ImageJ software [57]. Then, Pearson’s coefficient (PC) and the thresholded Mander’s coefficients tM_1_ and tM_2_ were calculated as described previously [33, 56].

### DNA purification and preparation of EPS extract

Genomic DNA of *B. subtilis* SBE1 (final concentration: 0.003 mol/L), *B. subtilis* 3610 (0.005 mol/L), *Shewanella oneidensis* MR1 (0.01 mol/L) and *Pseudomonas putida* KT2440 (0.07 mol/L) was extracted by using Wizard Genomic DNA Purification Kits (Promega). Exopolysaccharides (EPS) were extracted from batch cultures of *B. subtilis* SBE1 as previously described [7]. To remove the DNA, crude EPS was treated with both enzymes, followed by DNase I (100 μg mL^− 1^) for 1 h at 37 °C and proteinase K (100 μg mL^− 1^) for 1 h at 60 °C.

### Isothermal titration calorimetry (ITC)

Enthalpy changes (*∆H*) of the interaction between DNA and exopolysaccharides (EPS) were determined by isothermal calorimetry using a NANO ITC 2G (TA Instruments, USA). Exopolysaccharides and genomic DNA were dissolved in 0.01 M PBS (pH 7.4). Exopolysaccharides (2.25 mg mL^− 1^) were dispensed into the microcalorimetric cell (volume 1.3 mL), and the DNA solution was filled into the syringe compartment (volume 250 μL). DNA was titrated in 10 μL portions (3.14 μL for the first injection) into the EPS-containing cell under constant stirring, and the heat of reaction was plotted versus time. All measurements were performed at 25 °C. Data were analysed by using NANOANALYZE software [58].

### Statistical analysis

Data were basically described by means and respective standard deviations (SD). *P*-values were acquired using analysis of variance (ANOVA) followed by Tukey’s multiple comparisons test to evaluate statistical significance using SPSS 17.0 software. Differences were regarded as statistically significant when *p* < 0.05.

## Supplementary information

**Additional file 1 **Supplementary Information contains (1) additional details on the materials and methods; (2) a figure of structural arrangement of bacteria during biofilm development of *B. subtilis* SBE1 and its exopolysaccharide (EPS) mutant (∆*eps*G); (3) a figure of total carbohydrate content of EPS produced by *B. subtilis* SBE1 and its exopolysaccharide (EPS) mutant (∆*eps*G); (4) a figure of isothermal titration calorimetry measurement to determine the interaction of exopolysaccharide (EPS) and genomic DNA

## Data Availability

*Bacillus subtilis* SBE1 used in this study has been deposited in the China Centre for Type Culture Collection (CCTCC), and the accession number is CCTCC AB 2018210. The whole genome sequences of this strian have been deposited at DDBJ/ENA/GenBank under accession number QPGT01000000.The dataset supporting the conclusions of this article is included within the article (and its Additional files S1–3).

## Abbreviations

eDNA: Extracellular DNA
EPS: Exopolysaccharide
LTA: Lipoteichoic acid
LPS: Lipid polysaccharide
3D: Three-dimensional
HGT: Horizontal gene transfer
CCTCC: China Centre for Type Culture Collection
LB: Luria–Berta
MSgg: Minimal salt glycerol glutamate
CLSM: Confocal laser scanning microscopy
AFM: Atomic force microscopy
ITC: Isothermal titration calorimetry
*∆H*: Enthalpy changes
SD: Standard deviations
ANOVA: Analysis of variance
PC: Pearson’s coefficient
M1thr: Thresholded Mander’s tM_1_
M2thr: Thresholded Mander’s tM_2_
