# Supplementary material for: The exopolysaccharide–eDNA interaction modulates 3D architecture of Bacillus subtilis biofilm
Source: BMC Microbiol. 2020 May 14;20:115. doi: 10.1186/s12866-020-01789-5 (PMC7227074; doi:10.1186/s12866-020-01789-5)
Supplement: Supplementary file 1 — Additional file 1 Supplementary Information contains (1) additional details on the materials and methods; (2) a figure of structural arrangement of bacteria during biofilm development of B. subtilis SBE1 and its exopolysaccharide (EPS) mutant (∆epsG); (3) a figure of total carbohydrate content of EPS produced by B. subtilis SBE1 and its exopolysaccharide (EPS) mutant (∆epsG); (4) a figure of isothermal titration calorimetry measurement to determine the interaction of exopolysaccharide (EPS) and genomic DNA [file 12866_2020_1789_MOESM1_ESM.docx]

**Supporting information**

**The exopolysaccharide–eDNA interaction modulates 3D architecture of *Bacillus subtilis*** **biofilm**

Na Peng^1^, Peng Cai^1^*, Monika Mortimer^2^, Yichao Wu^1^, Chunhui Gao^1^, Qiaoyun Huang^1^

^1^State Key Laboratory of Agricultural Microbiology, College of Resources of Environment, Huazhong Agricultural University, Wuhan 430070, China

^2^Bren School of Environmental Science and Management and Earth Research Institute, University of California, Santa Barbara, California 93106, United States

*Corresponding author: Peng Cai

State Key Laboratory of Agricultural Microbiology

Huazhong Agricultural University, Wuhan, China

Phone: +86 27 87671033; Fax: +86 27 87280670

E-mail address: cp@mail.hzau.edu.cn

**MATERIALS AND METHODS**

**Phenol-sulfuric acid carbohydrate quantification**

The relative amounts of cell-associated carbohydrate were estimated as previously described [1, 2]. *B. subtilis* cells were grown in LB medium, then the cell concentration was adjusted to OD_650_ of 2.0 by adding the medium. The bacteria from 1ml normalized culture were collected by 14,500 × *g* for 5 minutes, and washed five times in 50 mM NaCl. Then the bacteria were re-suspended in 1 mL 50 mM EDTA, and incubated at 37 °C for 60 minutes. The cells were pelleted and the resultant supernatant was collected. The carbohydrate of supernatant was quantified as following: 30 µL of the sample, 30 µL of 5% phenol and 150 µL of sulfuric acid were mixed together in a 96-well microtiter plates (Costar, Corning Incorporated, Corning, NY). Color was allowed to develop for 30 minutes at room temperature, then measured at 490 nm in spectrophotometer. Concentration of carbohydrates were calculated from the standard curve. The sugar standards were diluted from a 50:50 mixture of 1 mg mL^-1^ sucrose and 1 mg mL^-1^ fructose.

[1] Brimacombe, C.A., Stevens, A., Jun, D., Mercer, R., Lang, A.S. and Beatty, J.T. Quorum-sensing regulation of a capsular polysaccharide receptor for the *Rhodobacter capsulatus* gene transfer agent (RcGTA). *Mol Microbiol* 87 (2013) 802-817.

[2] Byrd, M.S., Sadovskaya, I., Vinogradov, E., Lu, H.P., Sprinkle, A.B., Richardson, S.H., et al. Genetic and biochemical analyses of the *Pseudomonas aeruginosa* Psl exopolysaccharide reveal overlapping roles for polysaccharide synthesis enzymes in Psl and LPS production. *Mol Microbiol* 73 (2009) 622–638.


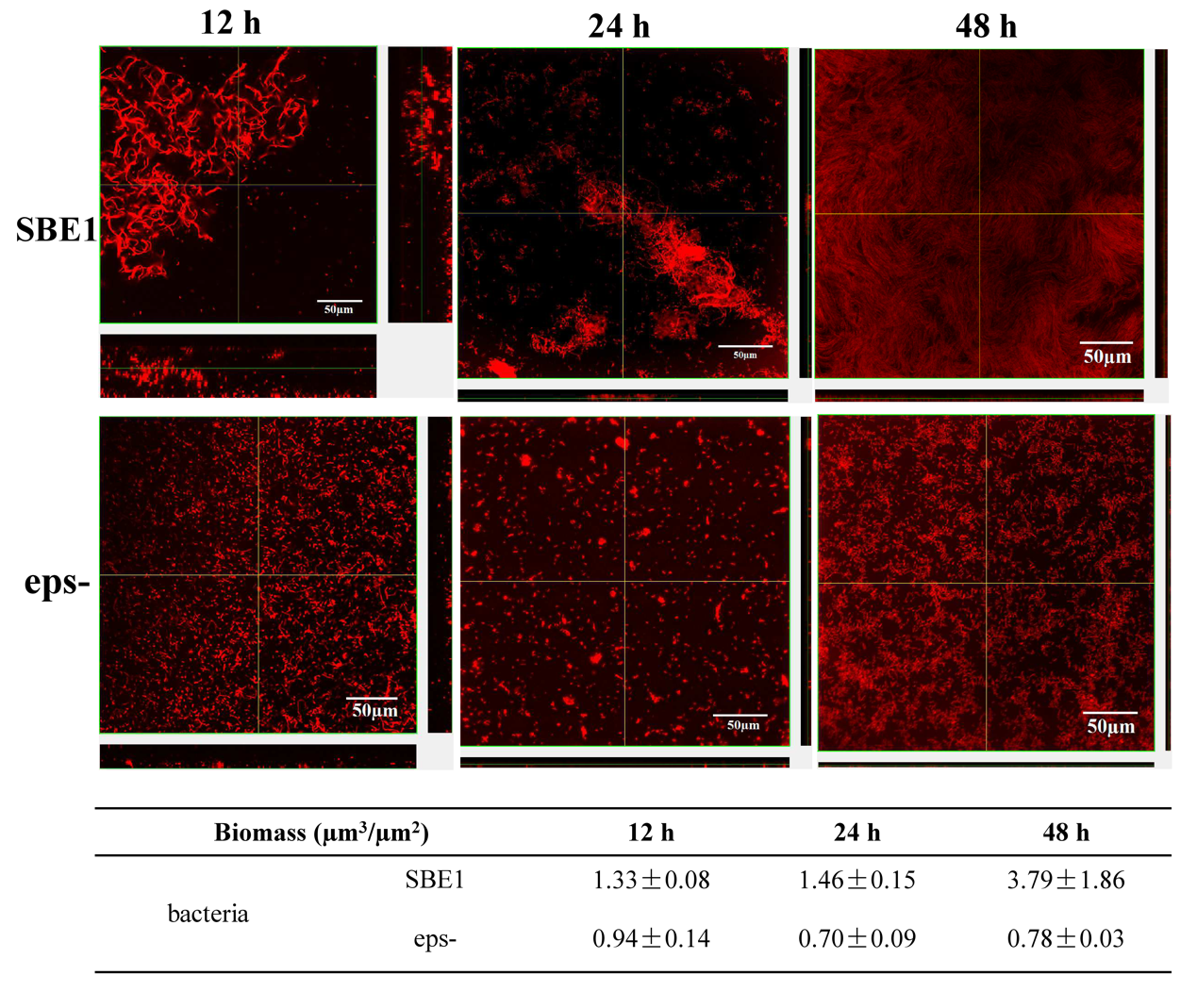


**Fig. S1** Structural arrangement of bacteria during biofilm development of *B. subtilis* SBE1 (upper panel) and its EPS mutant (*∆epsG*) (lower panel) at 12 h, 24 h and 48 h. The biomass values of bacteria in the biofilms were calculated using Imaris 7.4.2. The data shown are mean values ± SD (n = 3).


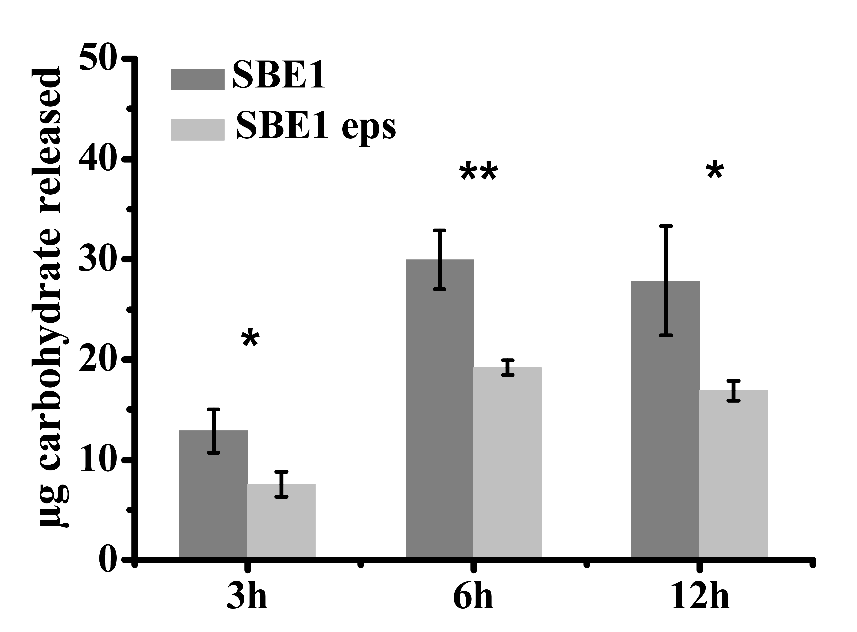


**Fig. S2** Total carbohydrate content of EPS produced by *B. subtilis* SBE1 and its EPS mutant (*∆epsG*). The data bars are means of three replicates and error bars represent standard deviations. ^*^*P* < 0.05, ^**^*P* < 0.01


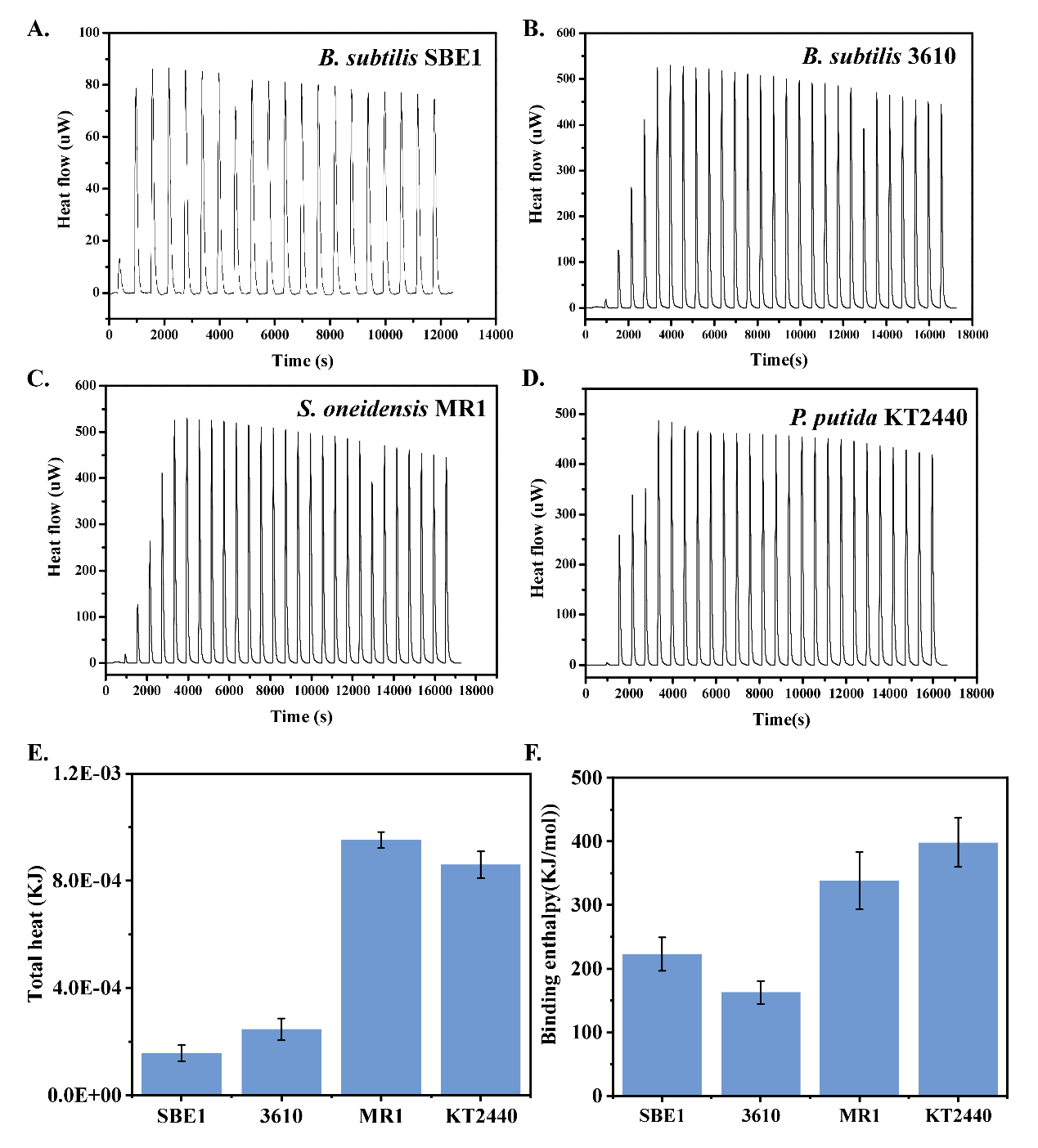


**Fig. S3** Isothermal titration calorimetry measurement to determine the interaction of EPS and genomic DNA of *B. subtilis* SBE1 (A), *B. subtilis* 3610 (B), *Shewanella oneidensis* MR1 (C) and *Pseudomonas putida* KT2440 (D). To genomic DNA of *B. subtilis* SBE1(0.003 mol L^−1^), *B. subtilis* 3610 (0.005 mol L^−1^), *Shewanella oneidensis* MR1 (0.01 mol L^−1^) and *Pseudomonas putida* KT2440 (0.07 mol L^−1^) of was titrated into EPS (2.25 mg ml^−1^) in 10 μl portions. (E) The total heat and (F) the calculated binding enthalpy for DNA binding to EPS (presented as mean value ± SD, n = 3).
